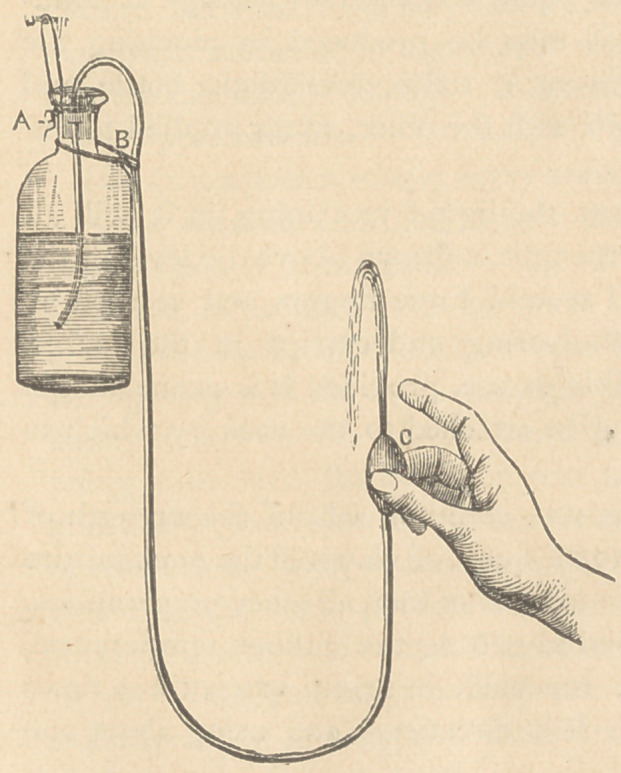# Nasal Catarrh

**Published:** 1870-07

**Authors:** Merritt F. Potter

**Affiliations:** Kaneville, Kane Co., Illinois


ARTICLE VI.—*Nasal Catarrh.* By MERRITT F. POTTER, M.D., Kaneville, Kane Co., Illinois.

The wide-spread prevalence and obstinate character of Nasal Catarrh, especially in the Northwest, seems almost to entitle it to a place among the "Opprobria Medicorum." The numerous remedies so conspicuously displayed in the newspapers, all claiming the merit of therapeutic infallibility, demonstrate both the extent and obstinacy of this disease. Consisting, as it does, of a chronic inflammation of the mucous membrane of the nasal cavities, the treatment required should be mainly local, and the failure to cure results not so much from ignorance of the proper remedy as from a want of a suitable method of applying it.

The practice of drawing medicines into the nasal cavities by inspiration is so extremely disagreeable as well as difficult, often strangling the sufferer, by entering the larynx, that the cures effected in this way are indeed "few and far between." The Catarrh Syringe is designed expressly for the topical medication of the nasal cavities, and doubtless many cases are greatly relieved by its use. But it should be used by the physician only, and requires so frequent and long continued use that many of those who adopt it for a while are soon tired of a course of treatment which requires so much time and labor. Besides, the liquid is thrown into the nasal cavities so rapidly and with so great force as to be extremely disagreeable to the patient, exciting sneezing and copious lachrymation, and failing to cleanse the morbid tissues because the liquid remains too short a time in contact with the affected part. The discovery by Prof. Weber, of Germany, in 1847, that liquid injected into one nostril through a closely fitting plug, would after filling the cavity, flow around the septum of the nose and escape from the other nostril without entering the throat, was of no ordinary importance, as foreshadowing a more rational system of therapeutics for this disease, and the subsequent invention by Prof. Thudichum of the device known as the Thudichum Douche, utilized the discovery of his German predecessor, and opened up a new avenue for the application of remedies to a region which, previously, had been nearly inaccessible to topical medication.

The advantages of this apparatus in treating morbid affections of the nasal organs, are very great. By its use it becomes an easy matter to thoroughly wash out the entire nasal cavity, completely deodorizing and disinfecting it, to remove all offensive secretions which accumulate in it, and to apply any remedial agent directly to the seat of the disease, arresting morbid action and disposing the diseased organ to take on the healing process. Valuable as this invention truly is, its bulk and liability to be broken operate to prevent its general adoption. What is wanted is an instrument compact and portable, so that it can be carried in the saddle-bags or pocket, and adapted for immediate use in any apartment without requiring special shelf room, or any fixtures not found in every dwelling. Such an apparatus seems to be represented in the accompanying cut.

C is a small hollow India Rubber bulb, open at both ends, and about the size and shape of a butternut. It is attached to one end of a small tube of the same material, about four feet in length. The other extremity of the tube is inserted in an ordinary pint or quart bottle, to which it may be attached in any convenient manner; a small elastic band looped around the tube and encircling the neck of the bottle, as shown in the figure, is sufficient. A flat piece of India Rubber, with a slotted opening through it barely large enough to allow the passage of the tube, and inserted in the neck of the bottle, will also retain the tube in place as shown in the cut. If the tube is passed through the loop by which the bottle is suspended, the tension of the loop will be sufficient to prevent the tube from slipping in ordinary cases. To use the instrument, it should be filled and suspended a little higher than the head, to a hook or a common nail. Next compress the bulb between the thumb and two middle fingers of one hand, so as to flatten it and bring the two opposite sides in contact. Press the index finger tightly over the outlet of the bulb and hold it there while the bulb is allowed to expand. The expansion of the bulb causes the liquid to flow through the syphon tube. The finger may then be removed and the bulb applied as a tight-fitting plug to one nostril. The liquid will flow into the nasal cavity of one side till it is filled, and then flowing around the septum of the nose it will be discharged by the other nostril. No possible danger of the liquid flowing into the Eustachian tube, or any other unpleasant effect, can ever result from this operation, provided the liquids are properly prepared and the patient be careful to adopt the simple precaution to *keep the mouth open* during the operation.

If a more forcible jet of the liquid is desired, to dislodge accumulations of adhering sordes, it may be produced by pinching the tube with one hand, to prevent a reflux toward the bottle, and forcibly compressing the bulb with the other, while applied to the nostril.

After using the instrument the liquid remaining in it will all flow back into the bottle if the tube is drawn above the level of the neck. It can then be coiled around three fingers and secured by an elastic band, or tied with a string and carried in the pocket. Any ordinary bottle is suitable to use, provided it is cleaned and a loop two or three inches long be attached to the neck by which to suspend it to a nail.

Of the various remedies in use, common salt, of the strength of about one and a half ounce to the quart of water, of the temperature of ninety-eight degrees, is of more value than all other preparations. Great care should be observed in getting the proper temperature, and the operation should be repeated, in severe cases, three times a day, passing the liquid in *both* directions, and using about one pint each time.

The bulb will fit a nostril of any size, and the material of which it is composed is secure against all danger from breakage. The instrument can scarcely be injured by any ordinary accident, and without any particular care in its usage will last for years.

MAY 10, 1870.

## Figures and Tables

**Figure f1:**